# Enhancing Business Intelligence by Means of Suggestive Reviews

**DOI:** 10.1155/2014/879323

**Published:** 2014-06-26

**Authors:** Atika Qazi, Ram Gopal Raj, Muhammad Tahir, Erik Cambria, Karim Bux Shah Syed

**Affiliations:** ^1^Faculty of Computer Science and Information Technology, University of Malaya, Lembah Pantai, 50603 Kuala Lumpur, Malaysia; ^2^Faculty of Information Science and Technology, COMSATS Institute of Information Technology (CIIT), Park Road, Islamabad 44000, Pakistan; ^3^Faculty of Computing and Information Technology, King Abdulaziz University, North Jeddah Branch, Jeddah 21589, Saudi Arabia; ^4^School of Computer Engineering, Nanyang Technological University, 50 Nanyang Avenue, Singapore 639798; ^5^Faculty of Business and Accountancy, University of Malaya, Lembah Pantai, 50603 Kuala Lumpur, Malaysia

## Abstract

Appropriate identification and classification of online reviews to satisfy the needs of current and potential users pose a critical challenge for the business environment. This paper focuses on a specific kind of reviews: the suggestive type. Suggestions have a significant influence on both consumers' choices and designers' understanding and, hence, they are key for tasks such as brand positioning and social media marketing. The proposed approach consists of three main steps: (1) classify comparative and suggestive sentences; (2) categorize suggestive sentences into different types, either explicit or implicit locutions; (3) perform sentiment analysis on the classified reviews. A range of supervised machine learning approaches and feature sets are evaluated to tackle the problem of suggestive opinion mining. Experimental results for all three tasks are obtained on a dataset of mobile phone reviews and demonstrate that extending a bag-of-words representation with suggestive and comparative patterns is ideal for distinguishing suggestive sentences. In particular, it is observed that classifying suggestive sentences into implicit and explicit locutions works best when using a mixed sequential rule feature representation. Sentiment analysis achieves maximum performance when employing additional preprocessing in the form of negation handling and target masking, combined with sentiment lexicons.

## 1. Introduction

The emergence of Web 2.0 technologies and the growing number of online reviews websites, such as Amazon, Epinions, and Cnet, emphasize user participation. People are encouraged to express their opinions/sentiments on purchased products. Sentiment analysis (also commonly referred to as opinion mining) is a natural language processing task that aims to track the public's mood regarding a particular product or service. This type of text analysis belongs to the field of natural language processing, computational linguistics, and text mining [1]. It is cumbersome and there is high time overhead for a human reader to find appropriate resources, extract opinion sentences, read, and then summarize them to obtain useful information. Thus, automated opinion detection and summarization systems are still required. Existing opinion mining approaches can be grouped into four main categories: keyword spotting, lexical affinity, statistical methods, and concept-level analysis [2]. Keyword spotting classifies text by effect categories based on the presence of unambiguous effect words such as happy, sad, afraid, and bored. Lexical affinity assigns arbitrary words a probable “affinity” to particular emotions. Statistical methods learn effective information by counting word cooccurrence frequencies from large annotated corpora [3–5]. Concept-level analysis consists in biologically inspired approaches that exploit the conceptual and effective information associated with multiword expressions (rather than single words) to infer emotional or polarity values from natural language opinions [6–8].

All opinion mining approaches are performed on reviews, which can be (1) regular or (2) comparative. The review types are differentiated based on language constructs that express diverse kinds of information [9]. Regular opinions pertain to a single entity only, and comparative opinions juxtapose two or more entities [9]. The study of these two sorts of opinions motivates the classification of more useful review types, such as suggestions. Where the suggestions are recently introduced as a third type of review in the study of opinion mining examined by Qazi et al. in [10]. Extracting suggestive sentences from text is valuable for numerous applications in the business, medical, and e-learning environments, among others. Clearly, suggestions on products/features are not only useful for product manufacturers, but also useful to potential customers who can better utilize products by keeping in view suggestions to avoid problems and take advantage of optimal product benefits.

Suggestions are indirect speech acts. Kumar [11] explains that speech acts meant to direct someone to do something through suggestions are classified as suggestive. Suggestions, on the other hand, are either (1) suggestive with expressed locution or (2) suggestive with implied locution [12]. The expressed suggestive forms are further split into two types: (1) explicit performatives and (2) implicit performatives, where explicit performatives are sentences expressed with performative verbs and implicit performatives are phrases that use “modals” to express the statement [13–15]. In the second type, that is, the implied suggestive, it can be said that reason or precondition depends upon the reader's inference. For instance, a simple opinion sentence about a person may be “Mr. X is very lazy.” An explicit phrase could be “I suggest using blue for better results,” while an implicit phrase may be “Let us go to the new café.” Few phrases are interrogative and imperative, and an example of an interrogative suggestive may be “Why doesn't she/he/it..?” and an imperative suggestive may refer to the “let us” suggestive.

Generally, suggestive sentences employ quite different language constructs from typical opinion sentences. Hereby, the aim is to study the issue of identifying suggestive sentences in text documents, for example, consumer reviews of products like movies and cell phones. The issue is challenging because although it is obvious that the above example sentences all contain some suggestive indicators, their semantic is not as suggestive as, “I could go to buy this phone.” Similarly, many sentences do not contain such indicators and are still suggestive sentences, like for instance “He might help you.” We first classify suggestive sentences into different categories based on existing linguistic research and then expand them into additional categories that are essential in practice. Subsequently, we propose a novel approach based on mixed sequences and supervised learning to identify suggestive sentences. A sequence of items is denoted as *S* = 〈*i*_1_, *i*_2_,…, *i*_*n*_〉, where every item corresponds to a feature that belongs to a certain token. The fundamental notion is a mixed sequence approach to achieve high recall and to build a supervised machine learning model to automatically classify each sentence into two classes: (1) suggestive and (2) nonsuggestive, in order to enhance precision. To sum up, the contributions made through this study are as follows: (1) propose a study of the problem with classifying suggestive sentences in text (to the best of our knowledge, there is no reported study on this matter so far); (2) categorize suggestive sentences into different types based on linguistic research; (3) perform sentiment analysis on classified suggestive reviews.

An effective approach to solve the drawback with feature construction using mixed sequences and the machine learning technology is thus proposed. This paper is organized as follows: in Section 2, related work is presented; Section 3 proposes the problem statement and categorizes different types of suggestive sentences, expanding on what is already available in linguistics; Section 4 presents the proposed technique; finally, Section 5 concludes the paper and suggests future directions.

## 2. Related Work

Related works from both computer science and linguistics are explored. Researchers in linguistics focus primarily on defining the syntax and semantics of suggestive constructs. Human communication is a broad phenomenon with multifarious facets and dimensions, including all signs whether textual, nontextual, verbal, and nonverbal. The majority of human communication occurs by means of utterances. According to linguists, suggestives are speech acts. The speech act theory was put forth by John Austin in his lectures published on how to do things with words [16]. Crystal [17] explained that according to Austin, there are two kinds of utterances, namely, constatives and performatives. Constatives are “statements that convey information” and performatives “do not communicate information, but are alike to actions,” examples of which include “I name this ship…” and “I promise…” [13].

Thus, language not only conveys information, but there are acts carried out or performed through words. Cook [18] elucidated that the speech act theory begins with the observation that there is a class of highly ritualistic utterances which carry no information about the world outside language at all, because they refer only to themselves. Examples of such declarations are swearing an oath, sentencing a criminal, opening a building, arresting a felon, or naming a ship. They are expressions in which saying the words and doing the action are the same thing: the function is created by the form. Searle [19] additionally contributed to this speech act theory. Crystal [17] further explained that there are direct speech acts and indirect speech acts, with suggestives falling in the indirect speech acts group. Indirect speech acts do not have an imperative form. Speech acts are observed to be addressing a listener directly, but most of them in mundane interactions are indirect [13, 17]. One instance is the variety of modes of requesting that someone carry out an action. “Close the widow,” for example, may carry a request in one situation and may convey a harsh tone to the listener.

Among the examples provided by Crystal to illustrate indirect speech acts are requests and suggestives. Consider the following example by Crystal: “It's getting cold in here, Shall we keep out the draught?” [17]. This speech act discussion given by Crystal shows that suggestives address the listener's “desire to perform the action or the speaker's reasons for having the action done.” Semantic analysis relies on logic which cannot be applied as a method of discerning suggestive reviews. The focus of these research work is on a limited set of suggestive constructs containing keywords like we/I suggest/propose, why does not he/she/it, ought to, if I were you, what is the wrong with, suppose, etc. In brief, although linguists have studied suggestive phrases and grammar, they are more for human use than for automatic identification of suggestive sentences by computers.

In text and data mining, no direct work on suggestive sentences has been identified so far. Sentiment classification and opinion extraction are closely related but differ from our work. To classify stock postings, a manually crafted lexicon in conjunction with several scoring methods was employed by Das and Chen [20]. Several supervised machine learning methods for sentiment classification of movie reviews were examined by Pang et al. [3]. A number of learning methods for review classification were additionally experimented with, which show whether classifiers perform better than a sentence on a whole review [21]. A study by [22] investigated sentence subjectivity classification. A method of finding adjectives indicative of positive/negative opinions was discussed. A similar technique with respect to nouns was also discussed by [23]. Other related works on sentiment classification and opinion detection, emotion detection have been carried out [22, 24–31]. Different unsupervised and supervised techniques were proposed to analyze opinions in customer reviews as well [5, 32]. Liu's work was later progressed by [33, 34]. Some literature is available on sentiment classification of comparative opinions. The difficulty with identifying comparative sentences and extracting comparative relations is addressed in some studies [9, 35–37]. From time to time, existing techniques are being explored and improved by researchers. However, none of these studies deal with suggestives, which is the focus of this work. To summarize, we are dealing with a new type of user input (suggestion) that is generally different from what is explored in existing studies. Such user input is valuable to users, potential buyers, and designers and is frequently available on e-commerce, blogs, and social media. Users are keen to share their experience with the public and also hope to learn from others' experience before they proceed with purchases. Hence, we argue the urgency of studying innovative types of reviews to aid product designers as well as customers in a wide range of helpful ways.

## 3. Study Approach

It is aimed via the work reported in this paper to fill a notable gap by offering designers and customers a possibility to process vast amounts of user input in the form of suggestive reviews. Initially, a linguistic view of suggestives is provided and some limitations are identified. They are then enhanced by classifying suggestive examples into suggestives with implicit and explicit locutions, as well as performing sentiment analysis on the suggestives.

### 3.1. Linguistic Perspective

As mentioned earlier in this discussion, suggestives are speech acts. More specifically, they are indirect speech acts. According to [11], speech acts suggesting someone to act in a particular way are known as suggestive. Most of suggestive sentence structures in English use the modal “should” which may imply a mild emphasis while communicating suggestion. Furthermore, the concept of suggestives becomes clearer once suggestives are compared with their contraries. Kumar maintained that directionals are the contraries of suggestives. The author explained that “the speech acts which are used to give direct instructions to someone to do something (as opposed to suggestives) are termed directional” [11]. Two broad types of suggestives have been identified: (1) expressed and (2) implied.

It is deduced that the opening keywords and phrases in suggestives are followed by infinitive verbs. Thus, constructions are should + infinitive, let us + infinitive, shall I + infinitive, do you want to + infinitive, why do not we/you + infinitive, we could/you could + infinitive, and ought to + infinitive. Clearly, most suggestive constructions follow a key suggestive word + infinitive verb structure. Nonetheless, there are exceptions, for example, I/we suggest/propose (suggest/propose that + subject + should + infinitive), if I were you, and what about/how about? (Key phrase (what about/how about) + gerund + noun). The above linguistic classification of suggestive sentences has two limitations: (1) nonsuggestive with suggestive words (in linguistics, sentences that contain suggestive indicators may not be used for identifying suggestive phrases, for example, in the phrase “In the context of cell phones, I did not suggest any one,” there is no suggestion being offered here); (2) limited coverage or suggestives with nonsuggestive words (in practice, there are many suggestive sentences that do not contain any of the above suggestive words; this limitation comes under an implicit type of suggestives).

### 3.2. Proposed Enhancements

To address the first limitation, computational methods will be used (i.e., machine learning) to distinguish suggestive from nonsuggestive examples. As for the second drawback, several feature sets and the way they are written in combinations of other words potentially indicating suggestions will be employed. The performance characteristics of additional knowledge will be portrayed besides the bag-of-words representation and experimenting with a different, more flexible representation called Mixed Sequential Rules that is evaluated within a machine learning framework. We also test the effect of feature selection. For sentiment lexicons, the effects of target masking, negation handling, and lexicon features are investigated.

## 4. Proposed Technique

In the present work, suggestives are studied at the sentence level. Thus, the problem is stated in view of sentences. Task (1) given a review,* r*, predicts its class,* c*, where* c* is a regular opinion, comparative or suggestive; task (2) given a suggestive review,* r*, predicts whether it is a suggestion with implicit or explicit locution; task (3) given a review,* r*, predicts whether its sentiment is a negative, neutral, or positive. As discussed earlier, a suggestive sentence is indicated both via specific keywords or more complex phrases and syntax patterns. Therefore, we have framed this problem as a supervised learning framework in an attempt to train a classification model capable of predicting whether a particular sentence contains a suggestion based on existing examples, which have already been classified as suggestions or not. In order to achieve superior classification performance and discover useful feature sets, we experiment with several combinations of feature sets and classification models. The feature sets are designed to accommodate existing state of the art in text classification, along with novel feature sets that utilize linguistic intuition behind suggestive statements. First, the various feature sets employed in this evaluation are described. However, suggestions may also exist without an explicit suggestion word. Similarly, nonsuggestive sentences can contain a suggestion word. In order to accommodate these nontypical cases, a machine learning approach that works on a wider feature space, looking beyond suggestive keywords, is applied. Such methods do not merely identify features that are indicative of a suggestive sentence, but also counter-indicative features, meaning they can indicate that a certain sentence is definitely not a suggestion.

### 4.1. Bag-of-Words Features

Since every statement in its raw form is represented as a sequence of words, it cannot be fed directly to the learning algorithms themselves, as most expect input in the form of real-valued feature vectors with a fixed size rather than raw text documents of variable length.

To address this, the following preprocessing approaches are used:

(i) *tokenizing* strings and giving an integer identifier for each possible token by using whitespaces and punctuation as token separators;
(ii) *transforming* the individual tokens for the purpose of data cleaning. In this step, all words are lowercased and stemming is applied via the Snowball stemmer [38];
(iii) *masking* the names of the review products, as well as the products they are being compared with. For this preprocessing technique the notation* “Msk” *is used. Any mention of the target product is replaced with “*[Target],*” and every mention of another product is replaced with* “[Other];”*
(iv) *negation processing*, transforming every word* w* in a window of length 2 after a negation word into a “not-*w*” token. With this technique, the notation* “Neg” *is used. Literature demonstrates that this particular preprocessing approach can improve sentiment analysis performance [39];
(v) *filtering* the tokens in order to remove words with low information value. All tokens which occur less than three times in the training set are filtered;
(vi) *counting* how many times word tokens occur in each document.

Features and samples are defined as follows.

(i) Each individual token occurrence frequency (normalized or not) is treated as a feature.
(ii) The vector of all the token frequencies for a given document is considered a Multivariate sample.

Description of documents depends upon word used in it with little or no care of where the word is used in the document. This follows that a corpus of documents can be depicted through a matrix form, whereby matrix *X* with a row for each document and column for each token (i.e., word) taking place in the corpus. The value of cell *x*_*ij*_ represents the number of times the word *j* appears in the example. In our evaluation, this feature set is denoted as “Bow” (bag-of-words). Since this approach is widely used in text classification, this feature set is deemed to be a baseline. Within preprocessing, part-of-speech tagging is performed as well, which is employed in other feature sets. The Maxent tagger, trained on the Penn Treebank features, is utilized to produce the part-of-speech tags [40].

### 4.2. Surface Features

This feature set covers peculiarities that may appear in informal texts, such as social media or public review boards. These features focus mainly on the style of expression and less on the content itself. This feature set is included to determine whether it carries any interesting information for suggestion classification:

- number of fully capitalized words in review “This phone is AWESOME” generates a value of 1.0;
- number of question words (how, why, what, who, when, and where) “Why and how is this broken?” generates a value of 2.0;
- number of negation words (not, never, neither, nobody, no, none, nor, nothing, and nowhere) which are words that negate meaning and can often affect the interpretation of the whole sentence, as revealed in the task of sentiment detection [20];
- number of contrast phrases (contrast, by the same token, conversely, instead, likewise, on one hand, on the other hand, on the contrary, rather, similarly, yet, but, however, still, nevertheless, and in contrast) which are words that signal the presence of a contrasting statement and often imply that the user is evaluating a certain object;
- number of question marks. In the evaluation, this feature set is denoted as “Surf.”

### 4.3. Suggestive Clues

Given the tokenized content annotated with part-of-speech tags, features are generated based on the presence of patterns given the theory described in Section 3. The tokenized and stemmed text is traversed and combined with its part-of-speech tag sequence to distinguish whether a pattern matches. If so, that feature's value is set to 1.0. For instance, the reviews “This phone is bad, I should have bought something else” and “If I were you, I'd stay away” produce the features {“should”: 1, “if_i_were_you”: 1}. We hypothesize that these patterns alone carry a large portion of information necessary for the successful detection of suggestions. This features set is denoted by “Sug.”

### 4.4. Comparison Clues

Comparator keywords were successfully combined with class sequential rules for the task of detective comparative sentences [41]. Since comparative sentences are a disjointed set from suggestive sentences, these features are also evaluated for suggestion extraction.

### 4.5. Performative Verbs

To take into account the background theory on how suggestions are presented, a lexicon of performative verbs is additionally included [21]. We hypothesize that including an indicator of a certain statement containing a performative verb may be indicative that the speaker is judging whether a product deserves a positive or negative suggestion. This feature set emits a single feature, with its value corresponding to how many performative verbs were detected in the example. This feature set is denoted as “Per.”

### 4.6. Sentiment Lexicon Features

Only for the sentiment analysis task, external knowledge is integrated in the current model by means of a sentiment lexicon. In particular, we use SenticNet, a knowledge base that maps a sentiment value from −1.0 to 1.0 to a set of words or multiword expressions [42]. The following features are generated:

- sum of all positive sentiments of all words;
- sum of all negative sentiments of all words;
- total objective sentiments of all words (where objective = 1.0 − (positive + negative)) score;
- ratio of total positive to negative scores for all words.

In addition to total sums, the same features for nouns, verbs, adjectives, and adverbs are also generated. Besides providing total sums, these features are also produced for nouns, verbs, adjectives, and adverbs separately. This feature is set as “Lex.”

### 4.7. Mixed Sequential Rules

A sequence of items is represented as *S* = 〈*i*_1_, *i*_2_,…, *i*_*n*_〉, where every item corresponds to a representation of a certain token. Recent methods for comparison classification have successfully used sequential patterns of part-of-speech tags surrounding certain keywords [9]. The researchers proposed a sequence construction method, which takes all sequences within a 3-token radius from keywords and replaces all the words except the keyword with their respective part-of-speech tag. To better explain the phenomenon, a method is herewith proposed that generates several features from the same token sequence, depending on representation level.

A mixed sequential rule feature generator is defined by two parts: a word of interest and its surroundings. Initially all token sequences are taken with lengths up to *n* that contain at least one word of interest. Then the feature is generated by combining the literal representation of the word of interest, and the surrounding tokens are represented by their respective part-of-speech tags. The following rules define various classes of words of interest for generating sequential rule features.

- Every verb is a word of interest. For instance 〈“buy,” “this,” “camera”〉 becomes “buy_DT_NP.”
- Every adjective is a word of interest. For instance 〈“buy,” “this,” “good” “camera”〉 becomes “VB_DT_good_NP.”
- Every adverb is a word of interest.
- Every suggestive keyword is a word of interest.
- Every comparative keyword is a word of interest.

Through this procedure, all subsequences of lengths up to 3 are generated. Each occurrence of a sequence rule in a given example generates a single feature corresponding to that sequence rule.

### 4.8. Feature Combination

In order to join the contribution of multiple feature sets, features are combined by performing a union of all features on every individual example. For instance, in a “Surf + Sug” feature construction setting for the example “This is an AWESOME phone, you should buy it!” the following would be generated: {“should”: 1.0 “fully_capitalized_words”: 1.0, “exclamation”: 1.0}. In the end, every known feature is enumerated and assigned a dimension in the vector space model, so that every column corresponds to a feature and every row corresponds to a review. The dataset is therefore represented as a matrix *X*, with *n* rows and *d* dimensions, where “*n*” is the number of examples and “*d*” is the number of known features in that particular feature combination.

### 4.9. Feature Selection

So as to reduce dimensionality and noisy features, applying the Chi-squared feature selection criterion [43] and maintaining the top 20% of the features are experimented with. The Chi-square statistic measures the lack of independence between the feature, *f*, and the class, *c*, given the training data which could be used to estimate extremeness based on Chi-square distribution with one degree of freedom. This feature selection approach selects only the features which are least likely to be class independent.

### 4.10. Learning Models

Supervised learning consists of learning the link between two datasets: data *X* and an external variable that we are trying to predict, usually called target or label and is denoted as *y*, a one-dimensional array of class values for the examples. Support vector machines using the linear kernel are employed for experimentation [44], as it has been shown to perform well in text classification tasks with many features. Logistic regression with *L*_1_ regularization also partakes in testing [21], and this has been shown to promote sparsity in the model, resulting in simpler models. Trials were done with different regularization parameter (the *C* parameter) values. For instance,* LR_C5* means that LR with *C* = 5.0 is applied.

**Figure 1:**
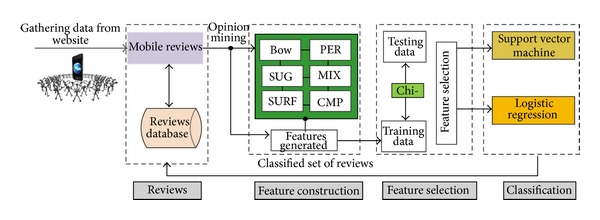
Suggestive and comparative detection workflow schematic.

The entire pipeline is visualized in Figure 1. First, the various feature construction methods are run on the input dataset, following which the various feature sets are combined into a common feature space, and feature selection is performed. Finally, the obtained single pruned feature space is used with a supervised machine learning model. This pipeline serves for both training the classification model and executing the actual predictions.

**Figure 2:**
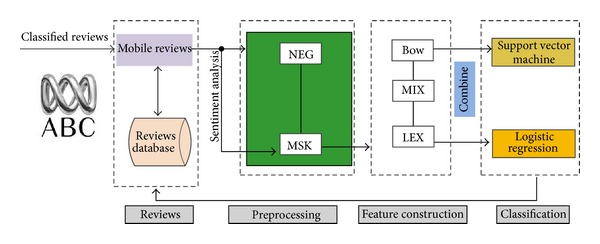
Sentiment analysis workflow schematic.

Figure 2 demonstrates a slightly different workflow pipeline. As some feature sets do not contribute significantly to performance, they are not present. The same applies to feature selection. On the other hand, the feature construction pipeline contains additional processing in the preprocessing step, namely, experimenting with the effect of negation handling and target masking.

### 4.11. Hypotheses

The baseline representation is a straightforward bag-of-words representation; one that we endeavor to improve. The hypotheses to be validated are:

- using suggestive key phrases improves performance;
- using comparison key phrases improves performance;
- using performative verb detection improves performance;
- using the mixed sequential rules representation improves performance.

## 5. Results and Discussion

### 5.1. Experimental Setting

With the intention of discovering the key features, several different feature set combinations are evaluated. Besides, several supervised learning algorithms and their hyper parameters such as the logistic regression chi-square (LRChi^2^), logistic regression (LR), standard vector machine chi-square (SVMChi^2^), and standard vector machine (SVM) are assessed. Each experiment is annotated by the feature sets and learning algorithm employed. The feature sets are marked with Bow, Cmp, Sug, Surf, and Mix. Where in case an experiment is marked with Bow + Cmp + Sug − SVM it means that its feature space is an amalgamation of bag-of-words features, comparison phrase indicator features and suggestive phrase indicator features, with SVM as the learning algorithm. These features are then transformed into a matrix representation and serve to train the learning model. The approach is evaluated on three classification setups:

- suggestive sentence detection: given an example, classify it as either suggestive or nonsuggestive;
- comparative sentence detection: given an example, classify it as either comparative or noncomparative;
- expressed/implied locutions detection: given an example suggestive sentence, classify it with either having expressed locution or implied locution.

The average, *F*_1_, precision and recall obtained on 10-fold cross-validation is reported. The dataset consists of 679 mobile phone reviews and is annotated with the type of review and its sentiment polarity. Out of the entire dataset, 181 are simple reviews, 189 are comparative reviews, and 309 are suggestive reviews. Of the suggestive reviews, 173 are suggestive with implicit locution, 100 with explicit locution, and 36 other suggestive reviews. The sentiment values were provided as star ratings ranging from 1 to 5 stars. One-star ratings were treated as negative, 2–4-star reviews were considered neutral, and 5-star reviews were considered positive.

### 5.2. Suggestive Example Detection

**Figure 3:**
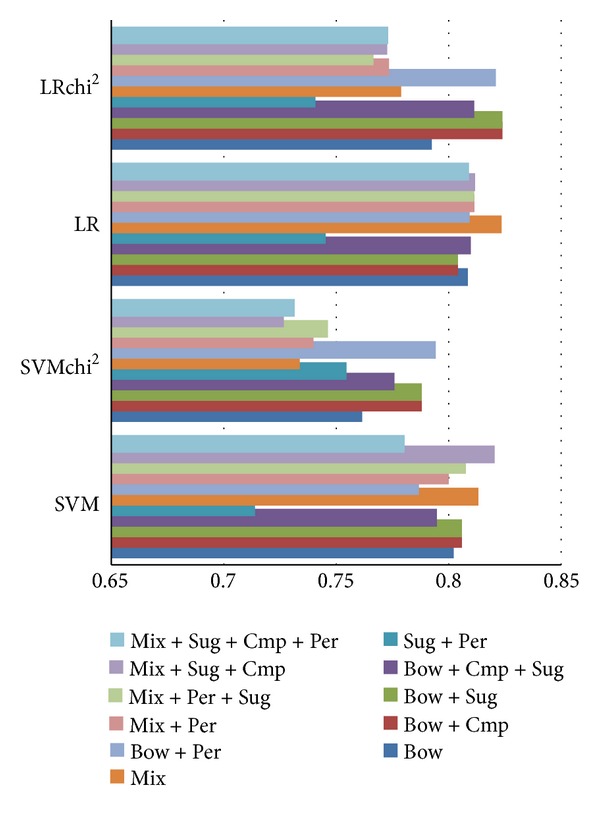
*F*_1_ performance on suggestive classification.

Pertaining to suggestive sentence detection, Figure 3 shows a small but statistically significant improvement in/after combining a bag-of-words feature set with suggestive clues. Comparative clues and surface features do not seem to discernibly benefit this task at all. Due to the presence of bag-of-words features, which results in large feature dimensionality, feature selection by choosing the 20% most important features additionally enhances performance.

By looking deeper into the relationship between precision and recall displayed in Table 1, it becomes obvious that while the bag-of-words baseline performs competitively, the combination of suggestive and comparison features offers a high-precision alternative with 91% precision.

### 5.3. Comparative Example Detection

**Figure 4:**
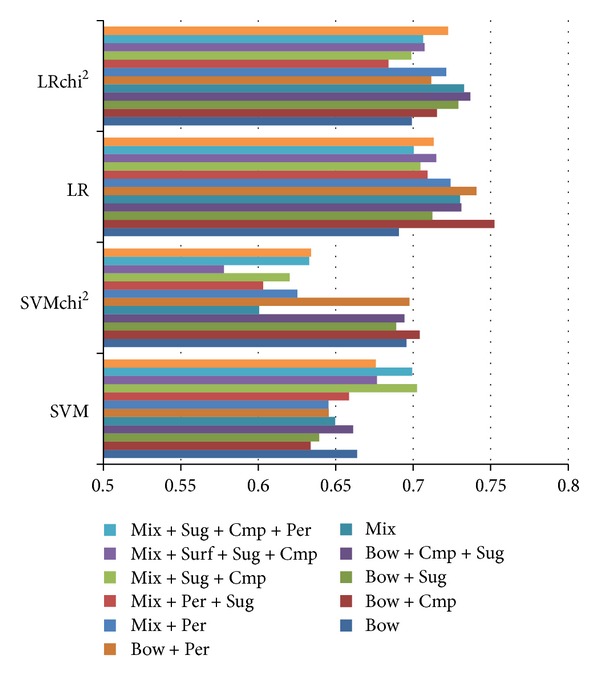
*F*_1_ performance on comparative classification.

Along the same lines, the issue of classifying comparative examples is also considered. It entails a disjointed set from the set of suggestive examples. The results have been obtained (see Figure 4 and Table 2). 

Figure 4 and Table 2 demonstrate that a bag-of-words model augmented by incorporating comparative features works best for detecting comparative reviews. Similar performance levels are also attainable by employing a lexicon of performative verbs or a mixed sequence model. Furthermore, it has been shown that feature selection does not improve performance.

### 5.4. Suggestive Example Classification into Explicit and Implicit Locutions

**Figure 5:**
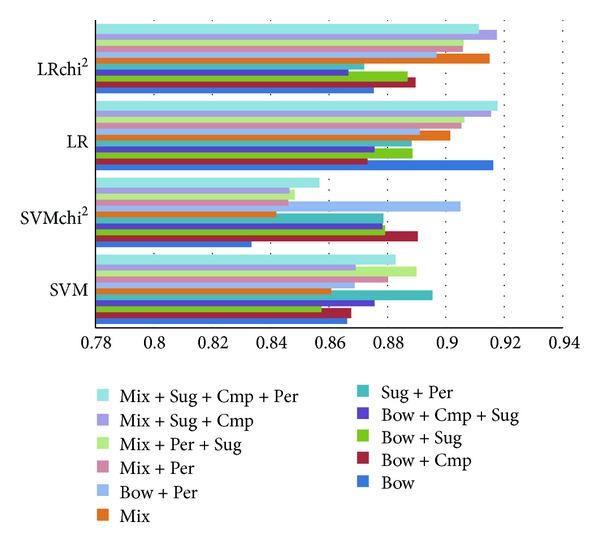
Performance numbers on sentiment analysis on suggestive sentences.

Figure 5 and Table 3 provide the performance details of various approaches. Here, the blend of suggestive keywords and performative verbs offers the simplest, best approach for distinguishing between implicit and explicit locutions. The same performance level was also achieved by the mixed sequence rules, especially in combination with suggestive and comparison patterns. This demonstrates that even without domain knowledge, such as performative verb lexicons, suggestive patterns or comparative patterns, one may still be able to well-perform by training a on a dataset, preprocessed by a bag-of-mixed-sequences approach.

### 5.5. Sentiment Analysis

**Figure 6:**
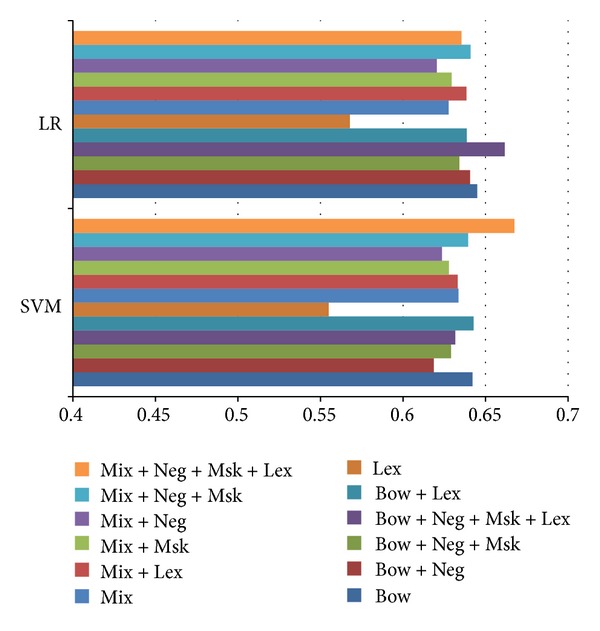
Performance numbers on various algorithms on separation of explicit and implicit locutions suggestions.

Figure 6 shows the performance details of sentiment analysis using various feature construction approaches. Here, the mixed sequences seem to make a good base feature set, capable of achieving adequate performance on its own and in some cases outperforming the bag-of-words representation. It is evident that the lexicons are insufficient when used alone, but they offer improved performance in combination with bag-of-words, masking, and negation handling. The finest performance was obtained with the mixed sequential rule base feature set using negation handling and target masking in preprocessing, combined with SenticNet lexicon features.

Table 4 portrays the precision-recall trade-offs of the evaluated feature sets, indicating that while individual preprocessing mechanisms are insignificant on their own, they add up to some improvement.

### 5.6. Feature Analysis

Once a linear model has been trained with training data, its coefficients of individual features for every binary classifier are noted. For a given binary classifier, it is possible to observe whether an individual feature is indicative of the class or counterindicative—in which case its presence puts it in another class. For the task of classifying suggestive sentences in a bag-of-words model, the features with the lowest weights (signals for nonsuggestive sentences) were* absolutely, company, better phone, prefer to, capture, functions, *and* ios*, while the highest weights (indicative of suggestive sentences) were* calendar, yet but, to text, fun, inches, not great, way of, let's, recently, *and* impressive*. While these features make a well-performing classifier, they simultaneously remain very typical for the general topic of phone reviews; few of the features would successfully generalize to other domains.

As for the weights of a model trained only on suggestive clues, almost all of the clues have positive weights, whereas only “*ought to*” has a slight negative weight. This confirms that the theory holds true in this dataset but that some of the rules may benefit from restricting their context. For instance, “*ought to” *can also be used in a 3rd person scenario, where the reviewer is describing a hypothetical aspect of a device and is not actually giving a suggestion.

With regard to the weights of a suggestion detection model consisting of mixed sequence rules, when looking at the set of features that indicate nonsuggestions, patterns such as* VB_good_., IN_more_than, charg_TO, CC_IN_was, should_be_ad, *and* realli_JJ* are observed. These indicate qualitative device descriptors. Conversely, the suggestions are specified by features such as* PRP_lock_RB, use_CC, will_need_TO,_go_RP, *and* catch_more_NN, *patterns that, interestingly, indicate device usage. Following that, people who suggest a device also tend to more vividly describe themselves as using it. For the problem of characterizing suggestive sentences by classifying them into implicit or explicit locution, the model's behavior when using suggestive and comparative features is noted. Comparators indicate implicit locution, and suggestive clues tend to indicative explicit locution more. This confirms the theory that when a reviewer explicitly recommends a product, they are likely to use more explicit terms, such as modal verbs. Upon viewing the same task on a different feature set, the best-performing mixed sequential rules, the pattern* VBZ_not, RB_worth_DT, *and* TO_use_CD *signifies implicit locution, while* forget_TO_put* and* camera_is_RB* are more indicative of explicit locution.

## 6. Conclusion and Future Work

A study of suggestive sentence classification and polarity detection was proposed in this paper. Different types of suggestive sentences were first analyzed from both linguistic and practical usage points of view. Aiming at identifying suggestive sentences, subsequent analysis was carried out through rule mining and machine learning approaches. The new approach was found effective by empirical evaluation based on mobile datasets.

Based on a comprehensive literature review and discussion, it can be argued that we spark a new debate on analyzing online product reviews. In order to construct models with outstanding performance in both suggestive and comparative tasks, the use of several feature sets was evaluated. Such feature sets may serve in detecting and characterizing suggestive sentences, and the combinations of various feature selection and supervised machine learning approaches were used. It was shown that for the task of detecting suggestive sentences, the basic bag-of-words model can be enhanced by augmenting it with additional features, especially with suggestive and comparative clues. The same approach also worked best in detecting comparative sentences. However, the bag-of-words representation may potentially lead to very topic-specific models, which may not translate to other domains, as indicated in the feature analysis. A combination of just suggestive and comparative keywords offers a high-precision alternative which can potentially also generalize better.

We would like to emphasize that, besides classifying suggestive reviews further in to explicit and implicit, we came across yet another novel type of reviews within this family. Although the finding of this type of review was not the main focus of this work, we have tentatively identified and reported it in this paper. Due to the lack of sufficient sample size (i.e., 36) we are cautious at this stage to classify these reviews separately within suggestive family. However, we do report this class for raising future research avenues based on larger sample size and deeper focused analysis on this type of reviews.

With respect to comparative sentence classification, no improvement was discerned when using a lexicon of performative verbs or a mixed sequential rule representation of the content. Nevertheless, it was demonstrated that in view of separating explicit and implicit locutions combining only the set of suggestive patterns with a lexicon of performative verbs offers optimal performance. Such performance level can be matched by training with mixed sequential rules. This indicates that flexible data representation is viable in instances where domain knowledge is unavailable. Since the dataset consists of a rather small number of examples, the hypothesis that feature selection is beneficial for both classification problems is confirmed, particularly when employing higher-dimensional representations based on the bag-of-words model. With regard to sentiment analysis, the mixed sequence features proved to be a promising base feature set and sometimes outperform the bag-of-words feature set. Overall, the best strategy for this type of dataset was to employ negation handling and target masking on the input text and augment the bag-of-words feature set with additional lexicon features. From the perspective of learning algorithms, both logistic regression and support vector machines perform comparably well in the three issues discussed before.

Big social data analysis [45] plays a key role in business intelligence, as manufacturers need to be able to efficiently process user feedback in order to enhance decision making and create business policies best capable of launching new products. In particular, more focused reviews (e.g., suggestive) are better able to increase satisfaction levels, leading to successful sales. In summary, more helpful reviews are introduced, such as making suggestions by classifying such sentences in online reviews. This sort of user input is valuable to designers as well as users, and it is becoming gradually more available with the rise of e-commerce and new social media including blogs and social networks. It has been remarked that where sentiment analysis is still required, mostly opinion mining and sentiment analysis present domain-dependent concepts that render purely syntactical approaches ineffective. Therefore, there is great need for common-sense computing techniques [46] so that the cognitive and affective gap between word-level natural language data and concept-level opinions can be bridged. To this end, future work will apply the same review types for concept-level sentiment analysis in order to explore the generalization of suggestion detection and characterization on other domains.

## Figures and Tables

**Table 1:** Performance numbers on LRChi^2^ over all feature construction approaches for detecting suggestive reviews.

Feature construction	Precision	Recall	*F*_1_
Bow	0.8806	0.7603	0.8129
Bow + Cmp	0.8797	0.7539	0.8093
Bow + Sug	0.8715	0.7668	0.8124
Bow + Cmp + Sug	0.8796	0.7733	0.8193
Bow + Cmp+ Sug + Surf	0.859	0.7572	0.8008
Sug	0.899	0.6535	0.7534
Mix	0.8388	0.7671	0.7975
Sug + Cmp	0.9126	0.6537	0.7568
Sug + Cmp + Surf	0.8937	0.6699	0.7586
Mix + Sug + Cmp	0.8271	0.78	0.8005
Mix + Surf + Sug + Cmp	0.8309	0.7703	0.7972

**Table 2:** Performance numbers on LR over all feature construction approaches for detecting comparative reviews.

Feature construction	Precision	Recall	*F*_1_
Bow	0.7297	0.6658	0.6907
Bow + Cmp	0.8298	0.7013	0.7523
Bow + Sug	0.7983	0.65	0.7124
Bow + Cmp + Sug	0.7821	0.6961	0.731
Sug + Per	0	0	0
Mix	0.7611	0.7132	0.7302
Bow + Per	0.8175	0.6842	0.7409
Mix + Per	0.7861	0.6789	0.724
Mix + Per + Sug	0.7347	0.6947	0.7092
Mix + Sug + Cmp	0.7377	0.6816	0.7047
Mix + Sug + Cmp + Per	0.7551	0.6618	0.7003

**Table 3:** Performance of LRChi^2^ on classification of explicit and implicit locution suggestions.

Feature construction	Precision	Recall	*F*_1_
Bow	0.878	0.876	0.875
Bow + Cmp	0.881	0.904	0.890
Bow + Sug	0.862	0.918	0.887
Bow + Cmp + Sug	0.843	0.896	0.867
Sug + Per	0.822	0.938	0.872
Mix	0.909	0.925	0.915
Bow + Per	0.901	0.897	0.897
Mix + Per	0.902	0.914	0.906
Mix + Per + Sug	0.913	0.903	0.906
Mix + Sug + Cmp	0.909	0.931	0.918
Mix + Sug + Cmp + Per	0.906	0.921	0.911

**Table 4:** Performance numbers on LR over all feature construction approaches for sentiment analysis of suggestive reviews.

Feature construction	Precision	Recall	*F*_1_
Bow	0.636	0.679	0.645
Bow + Neg	0.632	0.657	0.641
Bow + Neg + Msk	0.628	0.648	0.634
Bow + Neg + Msk + Lex	0.655	0.693	0.662
Bow + Lex	0.628	0.658	0.639
Lex	0.581	0.665	0.568
Mix	0.629	0.678	0.628
Mix + Lex	0.632	0.669	0.638
Mix + Msk	0.616	0.662	0.629
Mix + Neg	0.608	0.652	0.621
Mix + Neg + Msk	0.634	0.666	0.641
Mix + Neg + Msk + Lex	0.626	0.672	0.635
